# The prevalence of colistin resistance in clinical *Stenotrophomonas maltophilia* isolates worldwide: a systematic review and meta-analysis

**DOI:** 10.1186/s12866-023-02950-6

**Published:** 2023-07-28

**Authors:** Ali Delgarm Shams-Abadi, Abdollah Mohammadian-Hafshejani, David L. Paterson, Rezvan Arash, Elham Asadi Farsani, Asieh Taji, Hamid Heidari, Milad Shahini Shams Abadi

**Affiliations:** 1 Student Research Committee, Shahrekord University of Medical Sciences, Shahrekord, Iran; 2 Modeling in Health Research Center, Shahrekord University of Medical Sciences, Shahrekord, Iran; 3 UQ Center for Clinical Research, University of Queensland, Brisbane, Australia; 4 Cellular and Molecular Research Center, Basic Health Sciences Institute, Shahrekord University of Medical Sciences, Shahrekord, Iran; 5 International Campus, Shahid Sadoughi University of Medical Sciences, Yazd, Iran; 6 Department of Microbiology, Faculty of Medicine, Shahid Sadoughi University of Medical Sciences, Yazd, Iran

**Keywords:** Colistin resistance, *Stenotrophomonas maltophilia*, Systematic review, Meta-analysis

## Abstract

While trimethoprim-sulfamethoxazole (TMP-SMX) is the first-line therapy of *Stenotrophomonas maltophilia* infections, colistin is one of the therapeutic options in cases of allergy or resistance to TMP-SMX. However, understanding the global status of resistance to colistin amongst *S. maltophilia* isolates could be helpful for appropriate antibiotic prescription. This study aimed to conduct a systematic review and meta-analysis to examine the prevalence of colistin resistance in clinical *S. maltophilia* isolates worldwide. According to eligibility criteria, a total of 61 studies were included in the analysis. The pooled prevalence for colistin resistance was 42% (95% CI: 35-49%), ranging from 0.1 to 97%. Subgroups analysis indicated that, the pooled prevalence of colistin resistance was 44% (95% CI: 29-60%) in 15 studies during 2000–2010, and it was estimated to be 41% (95% CI: 33-50%) in 46 articles from 2011 to 2021. It was 46% (95% CI: 35-58%) in the studies that used broth microdilution method, and 39% (95% CI: 30-49%) in the studies with other used methods. The resistance rate in Asian countries was 45% (95% CI: 31-60%), in European countries was 45% (95% CI: 34-56%) and in the countries of North and South America was 33% (95% CI: 20-46%). Our review showed notable resistance to colistin in clinical *S. maltophilia* isolates. Given the estimated resistance rates, alternative antibiotics could be preferred to treat serious infections due to *S. maltophilia*.

**Supplementary Information:**

The online version contains supplementary material available at 10.1186/s12866-023-02950-6.

## Introduction

*Stenotrophomonas maltophilia* is a gram-negative non-fermenting bacillus that has been emerged as an important causative agent of severe hospital-acquired infections [1]. It causes several infections, such as bloodstream infection, secondary meningitis, and ventilator-associated pneumonia, predominantly amongst hospitalized patients [2].

Because of intrinsic antimicrobial resistance due to the presence of chromosomally encoded mechanisms, carbapenems and most beta-lactam antibiotics are ineffective against *S. maltophilia*. Acquired resistance through the horizontal acquisition of resistance genes or mutations, further limits therapeutic options for treating these challenging infections [3, 4]. In general, trimethoprim-sulfamethoxazole (TMP-SMX) is regarded as first-line therapy for *S. maltophilia* infections, and combination therapies with other antibiotics (e.g. levofloxacin or colistin) are alternative options in case of difficult-to-treat infections [5–8]. Hence, in the face of emerging resistance in gram-negative bacteria, global trends of colistin use are rising [8–10]. However, increased incidence of colistin-resistant *S. maltophilia* isolates has been recently described [11, 12]. Colistin resistance may occur through several mechanisms in Gram-negative bacteria. Mutations in the genes associated with LPS synthesis and modifications of this molecule are recognized mechanisms of the resistance. The expression of global genes could also be affected by environmental changes such as cations and pH variations. Furthermore, various phenotypic resistance mechanisms including adaptive resistance, heteroresistance and biofilm formation, accelerate the development of resistance [11].

There are various views on the effectiveness of colistin against *S. maltophilia* in literature [8–11]. Understanding the current global colistin resistance in this pathogen which is associated with high morbidity and mortality in chronic diseases and immunocompromised patients could be helpful for better perception of this issue, and appropriate prescription of antibiotic. To the best of our knowledge, there is no relevant comprehensive analysis. Therefore, the aim of the present study was to conduct a systematic review and meta-analysis to examine the prevalence of colistin resistance in clinical *S. maltophilia* isolates worldwide, conforms to the Preferred Reporting Items for Systematic Reviews and Meta-Analyses.

## Results

### Study characteristics

**Fig. 1:**
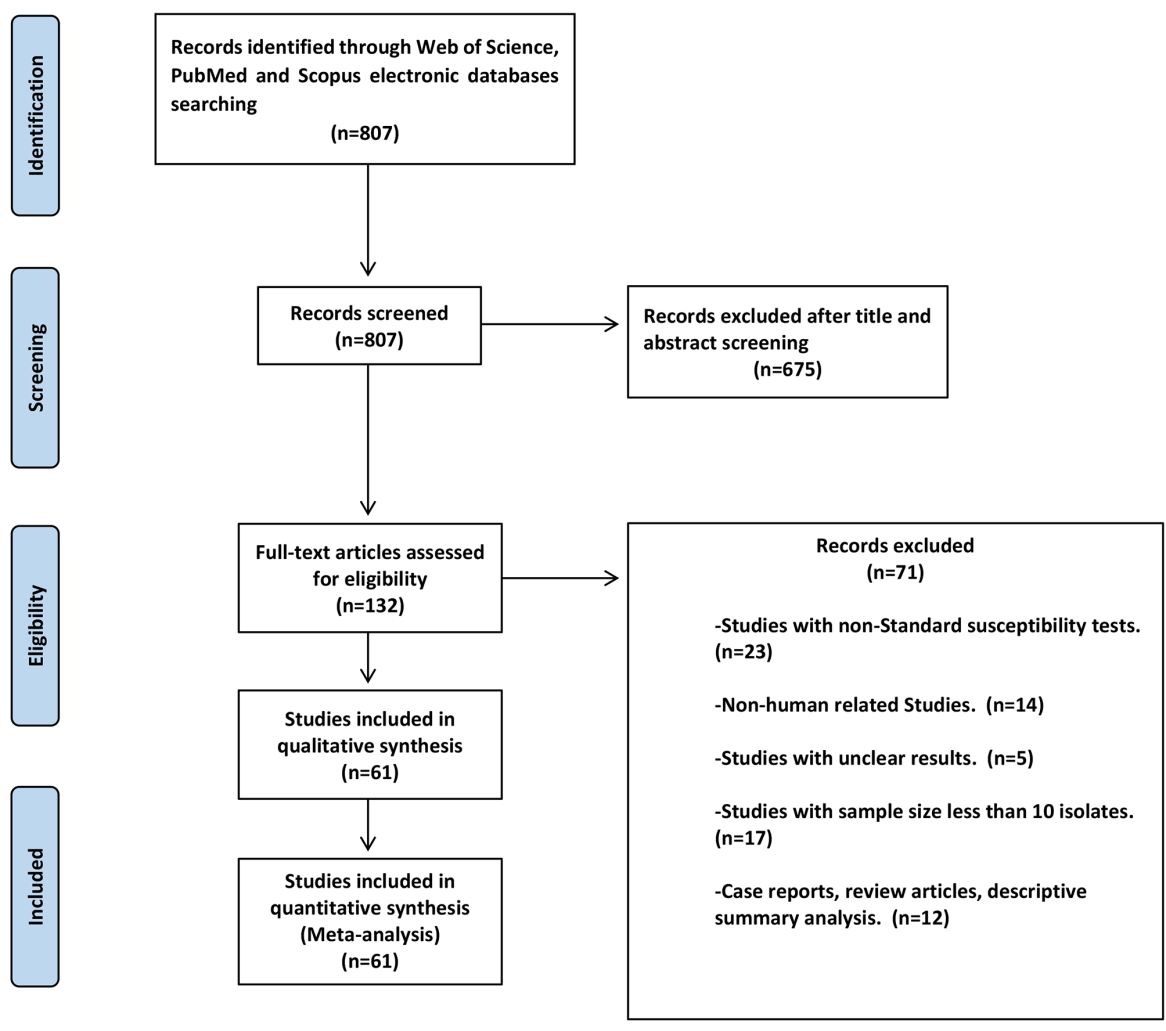
The flow chart of the selected studies

**Fig. 2:**
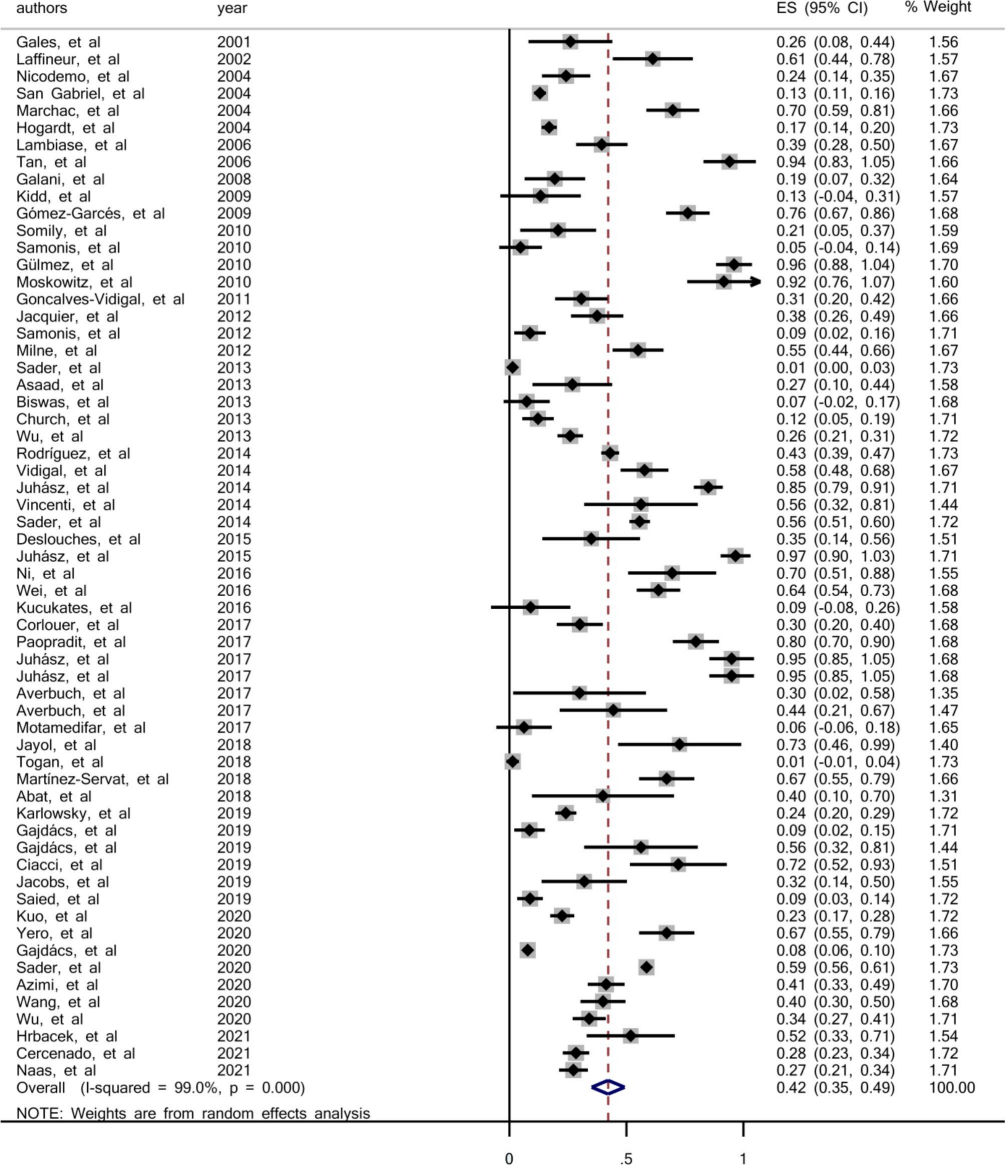
Forest plot for the prevalence of colistin resistance in clinical *S. maltophilia* during 2000–2021

As displayed in Fig. 1, a total of 807 articles were retrieved using the search strategy, 675 were excluded based on index and review of title and abstract, leaving 132 articles for full-text review. Full-text screening caused in exclusion of 71 more studies, resulting in 61 eligible studies. The main characteristics of the included studies and the prevalence of colistin resistance in clinical isolates of *S. maltophilia* are shown in Table 1. Sixty-one studies investigated the prevalence of colistin resistance in 9082 clinical isolates of *S. maltophilia*. From those studies, the pooled prevalence for colistin resistance in clinical isolates of *S. maltophilia* was 42% (95% confidence interval (CI): 35-49%), ranging from 0.1 to 97% (Fig. 2). The symmetric funnel plot showed no evidence of publication bias (Supplementary 1). There was no evidence of publication bias from Begg’s test (p = 0.597).

**Table 1:** Characteristics of studies included in the meta-analysis

Author	Year	Country	Continent	Sample size (N)	Colistin resistant (N)
Naas et al [13]	2021	France	Europe	201	55
Saied et al [14]	2020	France	Europe	102	9
Abat et al [15]	2018	France	Europe	10	4
Corlouer et al [16]	2017	France	Europe	83	25
Biswas et al [17]	2013	France	Europe	27	2
Jacquier et al [18]	2012	France	Europe	72	27
Cercenado et al [19]	2021	Spain	Europe	246	70
Martínez-Servat et al [11]	2018	Spain	Europe	61	41
Gómez-Garcés et al [20]	2009	Spain	Europe	80	61
Hrbacek et al [21]	2021	Czech	Europe	27	14
Yero et al [22]	2020	Europe	Europe	61	41
GAJDÁCS et al [23]	2020	Hungary	Europe	817	64
Gajdacs et al [6]	2019	Hungary	Europe	70	6
Gajdacs et al [24]	2019	Hungary	Europe	16	9
Juhász et al [12]	2017	Hungary	Europe	20	20
Juhász et al [25]	2017	Hungary	Europe	20	20
Juhász et al [26]	2015	Hungary	Europe	30	30
Juhász et al [27]	2014	Hungary	Europe	127	108
Ciacci et al [9]	2019	Italy	Europe	18	13
Vincenti et al [28]	2014	Italy	Europe	16	9
Lambiase et al [29]	2006	Italy	Europe	76	30
Togan et al [30]	2018	Turkey	Europe	72	1
Küçükates et al [31]	2016	Turkey	Europe	11	0
Gülmez et al [32]	2010	Turkey	Europe	25	24
Vidigal et al [33]	2014	Germany	Europe	90	52
Goncalves-Vidigal et al [34]	2011	Germany	Europe	65	20
Hogardt et al [35]	2004	Germany	Europe	506	86
Milne et al [36]	2012	Scotland	Europe	80	44
Samonis et al [37]	2012	Greece	Europe	68	6
Samonis et al [38]	2010	Greece	Europe	21	1
Galani et al [39]	2008	Greece	Europe	36	7
Marchac et al [40]	2004	England	Europe	63	44
Laffineur et al [41]	2002	Belgium	Europe	31	19
Kuo et al [42]	2020	Taiwan	Asia	253	57
Wu et al [43]	2021	Taiwan	Asia	170	58
Wang et al [44]	2020	Taiwan	Asia	100	40
Azimi et al [45]	2020	Iran	Asia	150	62
Motamedifar et al [46]	2017	Iran	Asia	16	0
Averbuch et al [47]	2017	Israel	Asia	18	8
Paopradit et al [48]	2017	Thailand	Asia	64	51
Wei et al [49]	2016	China	Asia	102	65
Ni et al [50]	2016	China	Asia	23	16
Asaad et al [51]	2013	Saudi Arabia	Asia	26	7
Somily et al [52]	2010	Saudi Arabia	Asia	24	5
Tan et al [53]	2006	Singapore	Asia	17	17
Deslouches et al [54]	2015	USA	North america	20	7
Church et al [55]	2013	USA	North america	90	11
Moskowitz et al [56]	2010	USA	North america	12	11
San Gabriel et al [57]	2004	USA	North america	673	88
Wu et al [58]	2013	Canada	North america	250	65
Rodríguez et al [59]	2014	Argentina	South America	641	276
Nicodemo et al [60]	2004	Brazil	South America	66	16
Gales et al [61]	2001	Brazil	South America	23	6
Kidd et al [62]	2009	Australia	Australia	15	2
Sader et al [63]	2020	Multi-country	Worldwide	1839	1078
Karlowsky et al [64]	2019	Multi-country	Worldwide	340	82
Jacobs et al [65]	2019	Multi-country	Worldwide	25	8
Jayol et al [66]	2018	Multi-country	Worldwide	11	8
Averbuch et al [67]	2017	Multi-country	Worldwide	10	3
Sader et al [68]	2014	Multi-country	Worldwide	494	275
Sader et al [69]	2013	Multi-country	Worldwide	362	5

### Subgroup analysis

**Fig. 3:**
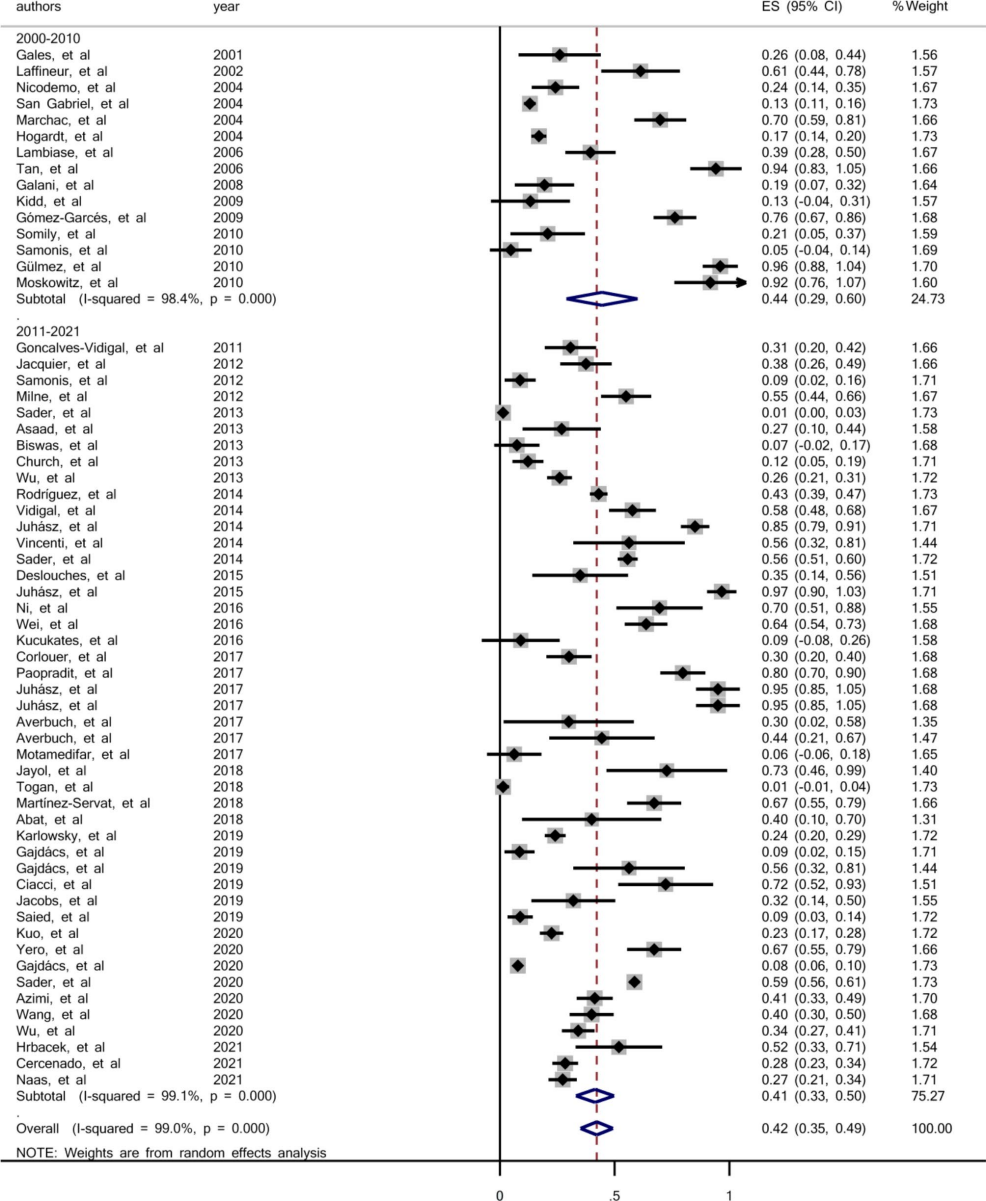
Forest plot for the prevalence of colistin resistance in clinical *S. maltophilia* during 2000–2010 and 2011–2021

To investigate the prevalence of colistin resistance in clinical isolates of *S. maltophilia* based on the study period, methods of susceptibility testing, geographical location, sample size, and quality assessment score of the articles, subgroup analysis was used. Based on this, the colistin resistance in clinical isolates of *S. maltophilia* during 2000–2010 was investigated in 15 studies, and the pooled prevalence was estimated 44% (95% CI: 29-60%) ranging from 0.5 to 96% (Fig. 3). There was a significant heterogeneity among the 15 studies (χ2 = 872.71; P < 0.001; I^2^ = 98.40). There was no evidence of publication bias from Begg’s test (p = 0.656). We found 46 articles that investigated the prevalence of colistin resistance in clinical isolates of *S. maltophilia* in 2011–2021. The pooled prevalence of colistin-resistant isolates was estimated 41% (95% CI: 33-50%), ranging from 0.1 to 97% (Fig. 3). Based on Q statistic and the I^2^ index heterogeneity was significant (χ2 = 4937.65; P < 0.001; I^2^ = 99.09%). There was no evidence of publication bias according to Begg’s rank correlation analysis (p = 0.925).

The prevalence of colistin resistance in clinical *S. maltophilia* isolates in the studies with a sample size equal to or less than one hundred samples was equal to 46% (95% CI: 35-57%), in the studies with a sample size of more than one hundred samples was equal to 33% (95% CI: 21-44%), in the studies in American countries was equal to 33% (95% CI: 20-46%), in the studies in Asian countries was equal to 45% (95% CI: 31-60%), in the studies in European countries was equal to 45% (95% CI: 34-56%) and in the studies that samples obtained from different continents was equal to 39% (95% CI: 12-66%) (Table 2).

**Table 2:** Subgroup analysis of the prevalence of colistin resistance in clinical *S. maltophilia* isolates

variables		Pooled prevalence [95% CI (%)]	No. of study	Range (%)	Heterogeneity		Publication bias (Begg’s test) -p-value		
					I^2^	P-value			
Overall		42 (35–49)	61	1–97	98.97	< 0.001		0.597	
Region	North and South America	33 (20–46)	8	12–92	97.20	< 0.001		0.458	
	Europe	45 (34–56)	33	1–97	98.84	< 0.001		0.299	
	Asia	45 (31–60)	12	6–94	95.96	< 0.001		0.337	
	Worldwide*	39 (12–66)	7	1–73	99.74	< 0.001		0.453	
	Australia	13 (0.04-38)	1	-	-	0.13		-	
Period	2000–2010	44 (29–60)	15	5–96	88.40	< 0.001		0.656	
	2011–2022	41 (33–50)	46	1–97	99.09	< 0.001		0.925	
Method	Broth microdilution	46 (35–58)	25	1–97	99.4	< 0.001		0.455	
	Other	39 (30–49)	36	1–96	97.8	< 0.001		0.687	
Sample size	One hundred and less	46 (35–57)	44	1–97	98.11	< 0.001		0.976	
	More than a hundred	33 (21–44)	17	1–85	99.5	< 0.001		0.026	
Quality of studies	Medium (4–6)	46 (35–57)	35	5–97	97.66	< 0.001		0.504	
	High (7–8)	37 (28–46)	26	1–95	99.27	< 0.001		0.193	

*Worldwide: Samples were from different countries on different continents

**Fig. 4:**
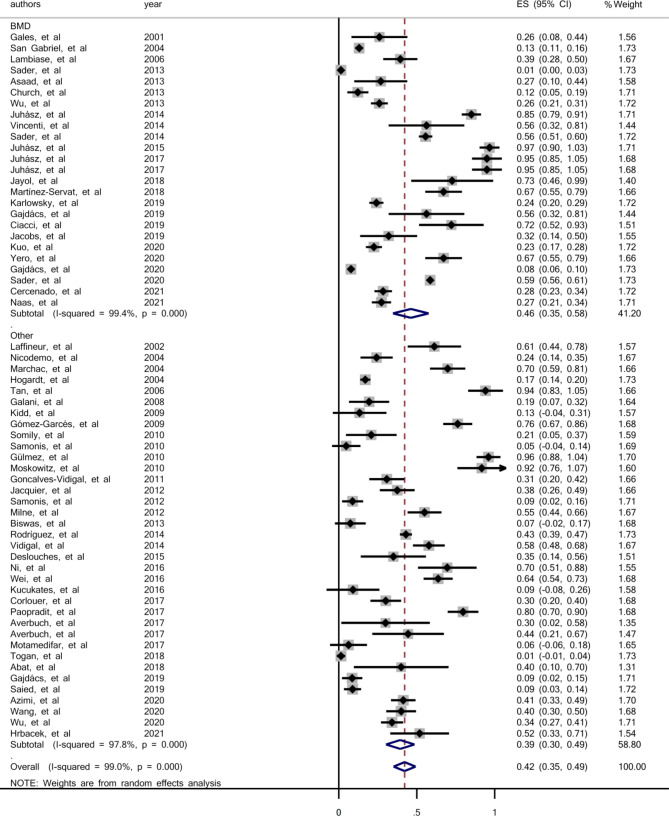
Forest plot for the prevalence of colistin resistance in clinical *S. maltophilia* based on used methods; BMD, Broth Microdilution Method; Other, other used methods

The prevalence of colistin resistance in the studies that used broth microdilution (BMD) was 46% (95% CI: 35-58%), and in the studies that used other methods, including DDM, agar dilution, E-test, VITEK 2 system and those without exact mentioned method was 39% (95% CI: 30-49%) (Fig. 4).

### Meta-regression and sensitivity analysis

In this meta-analysis, it was observed that the heterogeneity between the results of the studies is equal to 98.97%. To investigate the causes of heterogeneity, a meta-regression was performed in variables such as the year of the study, the sample size of the study, the quality evaluation score, the method of susceptibility testing and the geographical location of the study. The results from the meta-regression analysis determined there was no significant source of heterogeneity (*P* > 0.20). Moreover, sensitivity analysis was performed by excluding each study from the analysis one by one during each run. However, the final estimate of the prevalence of colistin resistance did not change significantly, which indicates the strength of the meta-analysis results (Supplementary 1).

## Discussion

*S. maltophilia* is intrinsically resistant to many antibiotics, such as penicillins, carbapenems and aminoglycosides and occurs in hospitalized patients, particularly in intensive care units (ICUs) [70–72]. *S. maltophilia* is not intrinsically resistant to polymyxins [73], hence, the present study aimed to systematically review the available scientific evidence regarding colistin resistance in clinical *S. maltophilia* isolates during the years 2000 to 2021. This systematic review is based on the published data spanning the globe.

According to our analysis, the pooled prevalence for colistin resistance among clinical *S. maltophilia* isolates was 42%. It was 44% from 2000 to 2010 and 41% in 2011–2021. Despite a slight reduction of colistin resistance in 2011–2021 studies compared to 2000–2010, there was no regular trend of colistin resistance among *S. maltophilia* clinical isolates during the period of 2000–2021.

In this study, the prevalence of colistin resistance in clinical isolates of *S. maltophilia* based on the antimicrobial susceptibility methods was also investigated. Challenges in the determination of susceptibility to colistin by laboratory testing are described frequently, and the most appropriate method is still controversial [74]. However, only MIC determination using broth microdilution method is recommended by the joint CLSI-EUCAST working group [75], and we believe it is the most appropriate. According to our analysis, the prevalence of colistin resistance in the studies which used the Broth Microdilution (BMD) method was 46% (95% CI: 35-58%), and in those studies which used other methods was 39% (95% CI: 30-49%). The obtained resistance rate by BMD was more than that by other methods. It seems that, determination of MIC and drug resistance by BMD method could lead to more accurate results and prescriptions of the antibiotic should be based on BMD results and not other methods.

In the present study, no study was included in the analysis from Africa, based on the eligibility criteria. The pooled prevalence of colistin resistance in clinical isolates of *S. maltophilia* in Europe, Asia and America was 45%, 45% and 33%, respectively. This rate was 39% for the included studies from the countries of different continents (Table 2). Although the same prevalence resistance to colistin in Europe and Asia was more than America, there was no notable difference between their resistance ranges (Table 2). Moreover, low number of studies were included and analyzed from America.

In the recent systematic review and meta-analysis regarding the global prevalence and distribution of levofloxacin, TMP/SMX, and minocycline resistance among clinical isolates of *S. maltophilia*, the rates of 14.4%, 9.2%, and 1.4% were reported, respectively [76]. These rates were lower than the estimated colistin resistance in the present study (42%). Therefore, these agents had better activity against *S. maltophilia* compared to colistin.

## Conclusions

The prevalence of colistin resistance in clinical isolates of *S. maltophilia* was estimated to be 42%. According to our analysis, this resistance rate has slightly decreased in the period 2011–2021 compared to 2000–2010. The prevalence of resistance in the studies using BMD method was also higher than that using other methods (46% vs. 39%). Given the toxicity of colistin, and the high prevalence of resistance of *S. maltophilia* to colistin, alternative antibiotics may be preferred for treating *S. maltophilia* infections.

## Methods

### Study details

In the present systematic review and meta-analysis study, all procedures relevant to the papers’ identification were carried out in accordance with the PRISMA (Preferred Reporting Items for Systematic Reviews and Meta-Analyses) Guidelines.

### Search strategy

To obtain all studies regarding the prevalence of colistin resistance in clinical isolates of *S. maltophilia*, a systematic search was done for English-language articles from January 1, 2000, to September 30, 2021, in the international databases PubMed/MEDLINE, Scopus and Web of Science. Records were managed by EndNote X9.0 software to exclude duplicates. The following MeSH terms were used simultaneously to find articles in databases: “*Stenotrophomonas maltophilia*’’, “*Stenotrophomonas*’’, “*maltophila*’’, “drug resistance”, and “antimicrobial resistance”. MeSH terms were combined with other words, including “*S. maltophilia*”, “colistin’’, “polymyxin’’, “antibiotic(s)” and their synonyms. To identify missing studies, we also searched bibliographies of retrieved articles for additional references.

### Eligibility criteria and study selection

Cross-sectional or cohort studies that reported the prevalence of colistin resistance in clinical isolates of *S. maltophilia* were considered. The titles, abstracts and full texts were screened independently by three reviewers (ADS, EAF and RA) to determine articles that met the inclusion criteria, and any discrepancies were resolved with a fourth investigator (MSSA) or by consensus. The articles published in English, indexed in PubMed/MEDLINE, Scopus and Web of Science with the following characteristics were included: reported the prevalence of colistin resistance in clinical isolates of *S. maltophilia* with standard laboratory tests. Studies were eligible if they had reported the prevalence of colistin resistance in *S. maltophilia.* Notably, the European Committee on Antimicrobial Susceptibility Testing (EUCAST) and Clinical and Laboratory Standards Institute (CLSI) do not provide breakpoints for colistin and *S. maltophilia*. Since the clinical isolates of this species possess high genetic diversity, and also the most reliable method to determine the activity of colistin against *S. maltophilia* is still controversial [11]. The laboratory tests for antibiotic susceptibility tests in the included studies were as follows; disk diffusion methods (DDM), minimum inhibitory concentration (MIC) determination by broth dilution, agar dilution and gradient strips, and the VITEK 2 system (bioMérieux). This study aimed to investigate the prevalence of colistin-resistant *S. maltophilia* isolates world wide. Studies were excluded if they did not report the prevalence of colistin resistance in clinical isolates of *S. maltophilia* or comment on the methods of susceptibility used. When the prevalence of colistin resistance from a given study was unavailable, or it was unclear if planned follow-up measurements were published, the authors requested this information via email. If the authors did not respond or did not provide the missing information, and if there was insufficient information available based on the publication, the study was excluded from the meta-analysis. We also excluded studies whose sample size was less than 10 isolates, nonhuman studies, studies published in languages other than English, review articles, meta-analyses or systematic reviews, congress abstracts and duplicate publications of the same study. Case reports were not included in the meta-analysis, as they do not have a denominator for any variables.

### Data extraction and definitions

Data collection was performed in parallel by three investigators who performed the literature search and also independently extracted the data from included studies. We extracted the following variables: first author’s name, the study performing time, publication date, the study setting, sample size (numbers of isolated *S. maltophilia*) and the prevalence of colistin and other antibiotic resistance.

### Quality assessment

The overall quality of studies was assessed using modified Critical Appraisal Checklist recommended by the Joanna Briggs Institute [77] and performed by two reviewers independently, and disagreements were resolved by discussion. The checklist is composed of eight questions that reviewers addressed for each study. The “Yes” answer for each question received a score of 1. Thus, the final scores for each study could range from 0 to 8. Two researchers independently assessed the quality of the articles, and discrepancies were discussed with a third researcher.

### Statistical analysis

In studies where the prevalence of colistin resistance in clinical isolates of *S. maltophilia* was calculated and presented separately for time or seasonal periods, using the meta-analysis method, a total prevalence of colistin resistance in clinical isolates of *S. maltophilia* was calculated from the presented values and considered in the analysis. Also, in studies where the prevalence of colistin resistance in clinical isolates of *S. maltophilia* was not reported, but the related data was available in the article text, the prevalence of colistin resistance was estimated. In the studies included in the meta-analysis, the presence of heterogeneity was assessed using graphical methods (forest plot) and statistical tests [chi-square test and I^2^ (heterogeneity quantification reporting)]. The heterogeneity of study results included in the meta-analysis was investigated using the chi-square test, and the type of design (fixed or random) was determined according to the test results [78, 79]. The most widely used measure of heterogeneity, I^2^, estimates the proportion of variability in a meta-analysis that is explained by differences between included studies rather than sampling error. Mathematically, I^2^ is expressed as I^2^ = τ^2^/ (σ^2^ + τ^2^), where τ^2^ represents the between-study heterogeneity, σ^2^ represents the total sampling error between studies, and σ^2^ + τ^2^ represents the total variance in the meta-analysis. A meta-regression model was used to identify factors associated with heterogeneity of results, accounting for study year, study sample size, quality score, susceptibility testing method, and geographic location. Sensitivity analysis was also used to assess the effect of omitting each study on the final result. Therefore, to determine the root of heterogeneity in the results of the studies included in the meta-analysis, subgroup analysis, sensitivity analysis, and meta-regression methods were used. Funnel diagrams and Egger’s tests were used for assessing publication bias. All analyses were performed by Stata statistical software (version 14.0, Stata Corp, College Station, TX), and the significance level in this study was considered < 0.05.

## Electronic supplementary material

Below is the link to the electronic supplementary material.

Supplementary Material 1

Supplementary Material 2

## Data Availability

The original contributions presented in the study are included in the article/Supplementary Material.

## Abbreviations

MDR: multidrug resistant
XDR: extensively drug resistant
PRISMA: Preferred Reporting Items for Systematic Reviews and Meta-Analyses
EUCAST: European Committee on Antimicrobial Susceptibility Testing
CLSI: Clinical and Laboratory Standards Institute
DDM: disk diffusion method
MIC: minimum inhibitory concentration
CI: confidence interval
ICUs: intensive care units
