# Supplementary material for: The prevalence of colistin resistance in clinical Stenotrophomonas maltophilia isolates worldwide: a systematic review and meta-analysis
Source: BMC Microbiol. 2023 Jul 28;23:200. doi: 10.1186/s12866-023-02950-6 (PMC10386657; doi:10.1186/s12866-023-02950-6)
Supplement: Supplementary file 1 — Supplementary Material 1 [file 12866_2023_2950_MOESM1_ESM.docx]

**Supplementary file 1:**

**
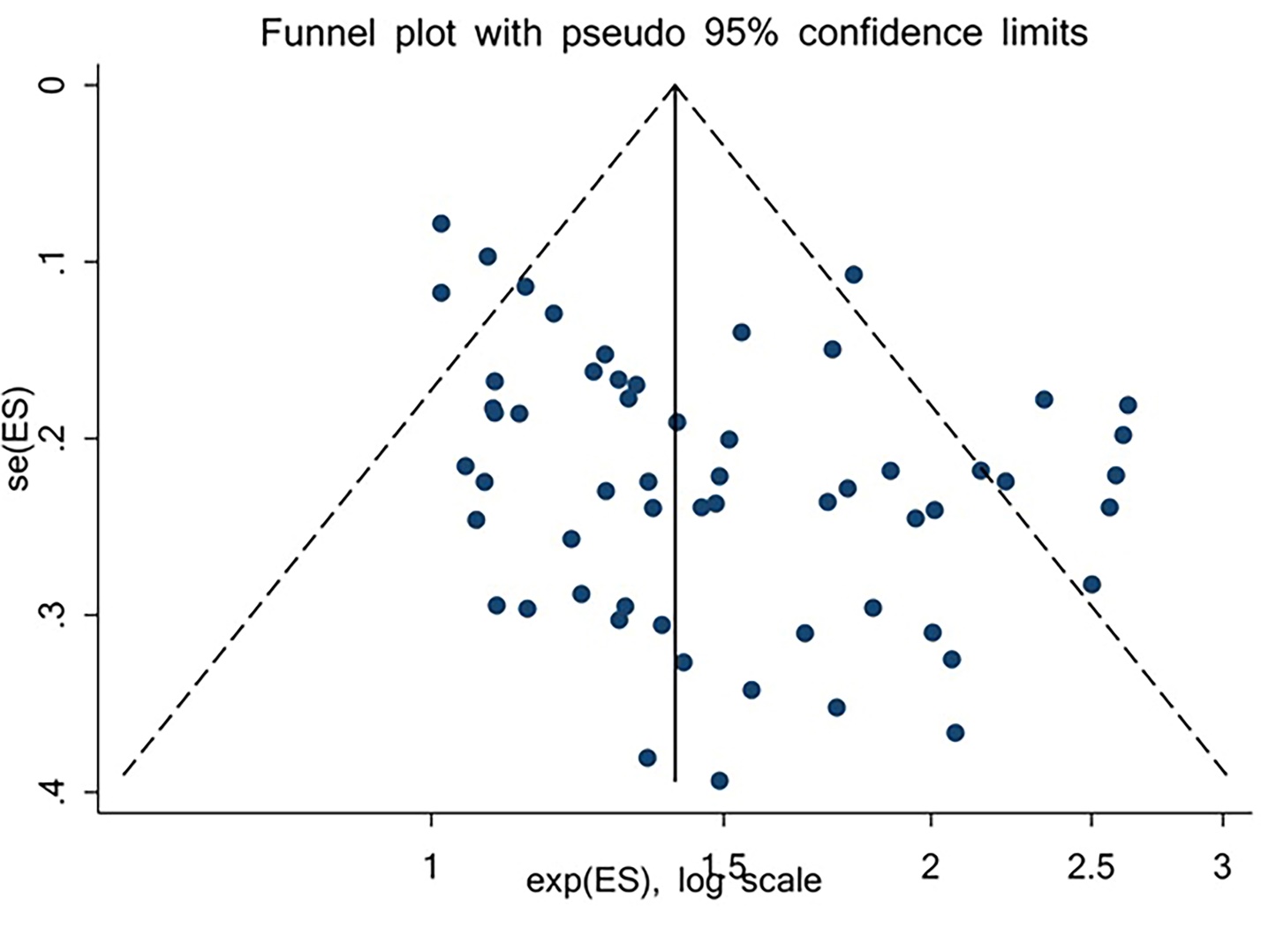
**

Supplementary figure 1. Funnel plot for studies included in the meta-analysis.

**Meta-regression and sensitivity analysis results**

A meta-regression was performed and included assessing the following variables: sample size, quality of study, study period, used method and geographical location. The results from the meta-regression analysis determined there was no significant source of heterogeneity (P > 0.20) (Supplementary table 1).

Supplementary table 1: Results of the Meta-regression analysis for the prevalence of colistin resistance in clinical *S. maltophilia* isolates obtained from hospitalized patients


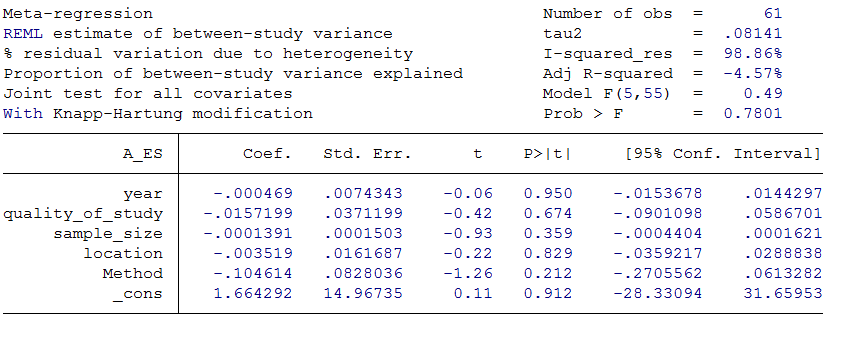


Also, sensitivity analysis was performed by excluding each study from the analysis one by one during each run. However, the estimated prevalence of colistin resistance in *clinical S. maltophilia* isolates obtained from hospitalized patients did not change significantly, further indicating the robustness of the meta-analysis results (Supplementary table 2, and figure 1).

Supplementary table 2: Results of the sensitivity analysis for the prevalence of colistin resistance in clinical *S. maltophilia* isolates obtained from hospitalized patients

| Study ID | * First author of the excluded article | Year | The estimated prevalence of colistin resistance in clinical *S. maltophilia* (95% CI) |
| --- | --- | --- | --- |
| 1 | Gales, et al | 2001 | 0.42(0.35-0.50) |
| 2 | Laffineur, et al | 2002 | 0.42(0.35-0.49) |
| 3 | Nicodemo, et al | 2004 | 0.43(0.35-0.50) |
| 4 | San Gabriel, et al | 2004 | 0.43(0.35-0.50) |
| 5 | Marchac, et al | 2004 | 0.42(0.35-0.49) |
| 6 | Hogardt, et al | 2004 | 0.43(0.35-0.50) |
| 7 | Lambiase, et al | 2006 | 0.42(0.35-0.49) |
| 8 | Tan, et al | 2006 | 0.41(0.34-0.48) |
| 9 | Galani, et al | 2008 | 0.43(0.35-0.50) |
| 10 | Kidd, et al | 2009 | 0.43(0.36-0.50) |
| 11 | Gómez-Garcés, et al | 2009 | 0.42(0.35-0.49) |
| 12 | Somily, et al | 2010 | 0.43(0.35-0.50) |
| 13 | Samonis, et al | 2010 | 0.43(0.36-0.50) |
| 14 | Guülmez, et al | 2010 | 0.41(0.34-0.48) |
| 15 | Moskowitz, et al | 2010 | 0.41(0.34-0.48) |
| 16 | Goncalves-Vidigal, et al | 2011 | 0.42(0.35-0.50) |
| 17 | Jacquier, et al | 2012 | 0.42(0.35-0.49) |
| 18 | Samonis, et al | 2012 | 0.43(0.36-0.50) |
| 19 | Milne, et al | 2012 | 0.42(0.35-0.49) |
| 20 | Sader, et al | 2013 | 0.43(0.36-0.50) |
| 21 | Asaad, et al | 2013 | 0.42(0.35-0.50) |
| 22 | Biswas, et al | 2013 | 0.43(0.36-0.50) |
| 23 | Church, et al | 2013 | 0.43(0.36-0.50) |
| 24 | Wu, et al | 2013 | 0.42(0.35-0.50) |
| 25 | Rodríguez, et al | 2014 | 0.42(0.35-0.49) |
| 26 | Vidigal, et al | 2014 | 0.42(0.35-0.49) |
| 27 | Juhász, et al | 2014 | 0.41(0.35-0.48) |
| 28 | Vincenti, et al | 2014 | 0.42(0.35-0.49) |
| 29 | Sader, et al | 2014 | 0.42(0.35-0.49) |
| 30 | Deslouches, et al | 2015 | 0.42(0.35-0.50) |
| 31 | Juhász, et al | 2015 | 0.41(0.34-0.48) |
| 32 | Ni, et al | 2016 | 0.42(0.35-0.49) |
| 33 | Wei, et al | 2016 | 0.42(0.35-0.49) |
| 34 | Kucukates, et al | 2016 | 0.43(0.36-0.50) |
| 35 | Corlouer, et al | 2017 | 0.42(0.35-0.50) |
| 36 | Paopradit, et al | 2017 | 0.42(0.35-0.49) |
| 37 | Juhász, et al | 2017 | 0.41(0.34-0.48) |
| 38 | Juhász, et al | 2017 | 0.41(0.34-0.48) |
| 39 | Averbuch, et al | 2017 | 0.42(0.35-0.49) |
| 40 | Averbuch, et al | 2017 | 0.42(0.35-0.49) |
| 41 | Motamedifar, et al | 2017 | 0.43(0.36-0.50) |
| 42 | Jayol, et al | 2018 | 0.42(0.35-0.49) |
| 43 | Togan, et al | 2018 | 0.43(0.36-0.50) |
| 44 | Martínez -Servat, et al | 2018 | 0.42(0.35-0.49) |
| 45 | Abat, et al | 2018 | 0.42(0.35-0.49) |
| 46 | Karlowsky, et al | 2019 | 0.43(0.35-0.50) |
| 47 | Gajdács, et al | 2019 | 0.43(0.36-0.50) |
| 48 | Gajdács, et al | 2019 | 0.42(0.35-0.49) |
| 49 | Ciacci, et al | 2019 | 0.42(0.35-0.49) |
| 50 | Jacobs, et al | 2019 | 0.42(0.35-0.49) |
| 51 | Saied, et al | 2019 | 0.43(0.36-0.50) |
| 52 | Kuo, et al | 2020 | 0.43(0.35-0.50) |
| 53 | Yero, et al | 2020 | 0.42(0.35-0.49) |
| 54 | Gajdács, et al | 2020 | 0.43(0.35-0.50) |
| 55 | Sader, et al | 2020 | 0.42(0.35-0.49) |
| 56 | Azimi, et al | 2020 | 0.42(0.35-0.49) |
| 57 | Wang, et al | 2020 | 0.42(0.35-0.49) |
| 58 | Wu, et al | 2021 | 0.42(0.35-0.49) |
| 59 | Hrbacek, et al | 2021 | 0.42(0.35-0.49) |
| 60 | Cercenado, et al | 2021 | 0.42(0.35-0.50) |
| 61 | Naas, et al | 2021 | 0.42(0.35-0.50) |
| Combined | | | 0.42(0.35-0.49) |

* The first author of the excluded article in each phase of analysis and calculation of the prevalence of colistin resistance in clinical *S. maltophilia*


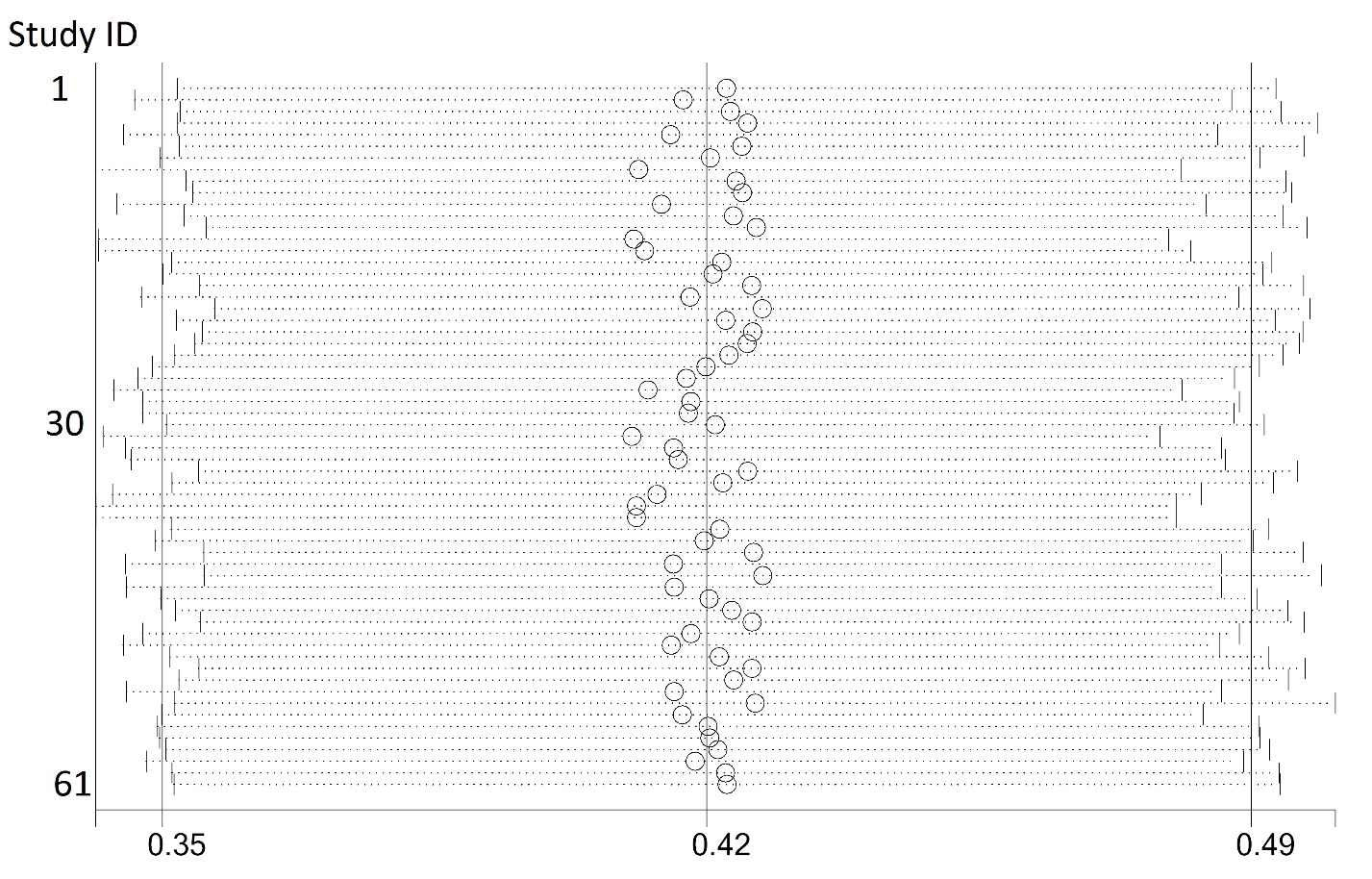


Supplementary figure 2: Results of the sensitivity analysis for the prevalence of colistin resistance in clinical *S. maltophilia* isolates obtained from hospitalized patients
